# Supplementary material for: A Likelihood Approach for Real-Time Calibration of Stochastic Compartmental Epidemic Models
Source: PLoS Comput Biol. 2017 Jan 17;13(1):e1005257. doi: 10.1371/journal.pcbi.1005257 (PMC5240920; doi:10.1371/journal.pcbi.1005257)
Supplement: S1 File — (TAR.GZ) [file pcbi.1005257.s014.tar.gz › HSPH_Online-SI-Revision/IPoi09/n10-severe/n10-severe_BENCH2_table0.pdf]

1	newly 3 weeks spec	422.	517.667	{318., 816.}	36.1581	0.129904	newly 3 weeks cumul	1572.	1787.62	{1210., 2444.}	25.7018	0.100459	newly 5 weeks spec	319.	364.286	{182., 652.}	44.3997	0.167983	newly 5 weeks cumul	2230.	2593.57	{1597., 3955.}	30.5737	0.116121	newly 51 weeks cumul	3236.	3955.52	{2248., 6358.}	38.7427	0.137391
2	newly 3 weeks spec	559.	659.677	{443., 888.}	30.6134	0.121165	newly 3 weeks cumul	1850.	2187.24	{1609., 2751.}	29.3505	0.124695	newly 5 weeks spec	307.	494.419	{280., 755.}	64.8524	0.196757	newly 5 weeks cumul	2596.	3254.31	{2253., 4206.}	32.7098	0.123037	newly 53 weeks cumul	3551.	5280.	{3214., 7748.}	52.5077	0.168935
3	newly 3 weeks spec	372.	762.984	{568., 962.}	105.103	0.30265	newly 3 weeks cumul	1462.	2519.82	{1830., 3037.}	73.2782	0.231453	newly 5 weeks spec	176.	557.066	{317., 957.}	216.515	0.468845	newly 5 weeks cumul	1920.	3733.25	{2772., 4555.}	94.4399	0.28217	newly 55 weeks cumul	2258.	5687.97	{3753., 8561.}	151.903	0.385056
4	newly 3 weeks spec	431.	505.121	{259., 748.}	37.959	0.157251	newly 3 weeks cumul	1668.	1678.78	{961., 2305.}	25.8073	0.130343	newly 5 weeks spec	272.	380.931	{198., 618.}	56.8839	0.189439	newly 5 weeks cumul	2316.	2502.28	{1338., 3569.}	30.7412	0.13615	newly 50 weeks cumul	2990.	4310.03	{2397., 6887.}	56.2311	0.182271
5	newly 3 weeks spec	395.	505.121	{259., 748.}	43.8193	0.167254	newly 3 weeks cumul	1512.	1678.78	{961., 2305.}	29.8588	0.136121	newly 5 weeks spec	274.	380.931	{198., 618.}	56.2673	0.188562	newly 5 weeks cumul	2125.	2502.28	{1338., 3569.}	35.2552	0.143451	newly 50 weeks cumul	3018.	4310.03	{2397., 6887.}	55.3895	0.180875
6	newly 3 weeks spec	500.	611.155	{372., 773.}	35.169	0.156594	newly 3 weeks cumul	1731.	1904.74	{1348., 2327.}	23.5831	0.131298	newly 5 weeks spec	216.	505.121	{259., 748.}	137.38	0.355882	newly 5 weeks cumul	2283.	2972.36	{1963., 3663.}	39.8848	0.165555	newly 51 weeks cumul	2710.	5603.62	{3638., 7968.}	107.011	0.295648
7	newly 3 weeks spec	266.	449.305	{222., 699.}	74.6973	0.224791	newly 3 weeks cumul	943.	1586.92	{896., 2227.}	72.7951	0.226847	newly 5 weeks spec	142.	306.881	{140., 515.}	119.718	0.29923	newly 5 weeks cumul	1279.	2266.36	{1224., 3529.}	80.1341	0.236338	newly 50 weeks cumul	1805.	3408.63	{1708., 5526.}	92.6597	0.253283
8	newly 3 weeks spec	348.	708.452	{446., 1003.}	103.586	0.29013	newly 3 weeks cumul	1582.	2402.01	{1690., 2897.}	52.8263	0.176145	newly 5 weeks spec	203.	481.37	{258., 800.}	137.195	0.339865	newly 5 weeks cumul	2074.	3475.63	{2322., 4581.}	68.013	0.212849	newly 54 weeks cumul	2481.	5115.75	{3124., 7556.}	106.197	0.292623
9	newly 3 weeks spec	571.	924.429	{679., 1175.}	63.5699	0.206514	newly 3 weeks cumul	2047.	3099.48	{2347., 3606.}	55.8386	0.196548	newly 5 weeks spec	361.	615.635	{372., 977.}	72.5498	0.209383	newly 5 weeks cumul	2908.	4477.08	{3485., 5367.}	54.9453	0.185557	newly 58 weeks cumul	3573.	6230.22	{4526., 8529.}	74.3695	0.229932
10	newly 3 weeks spec	479.	688.26	{336., 1061.}	57.3798	0.184502	newly 3 weeks cumul	1759.	2730.3	{1535., 3836.}	59.5193	0.189509	newly 5 weeks spec	209.	336.123	{139., 574.}	74.628	0.214689	newly 5 weeks cumul	2339.	3562.95	{1957., 5391.}	58.592	0.185327	newly 58 weeks cumul	2666.	4228.05	{2241., 6541.}	65.0741	0.198155
11	newly 3 weeks spec	358.	894.242	{617., 1176.}	149.788	0.3858	newly 3 weeks cumul	1919.	2950.56	{2279., 3469.}	54.8547	0.185188	newly 5 weeks spec	90.	594.403	{328., 949.}	560.448	0.783897	newly 5 weeks cumul	2152.	4294.92	{3240., 5236.}	99.578	0.292969	newly 57 weeks cumul	2254.	5951.85	{4007., 7983.}	164.057	0.407494
12	newly 3 weeks spec	587.	924.718	{682., 1141.}	59.6348	0.198803	newly 3 weeks cumul	1997.	2977.96	{2506., 3356.}	52.0541	0.18474	newly 5 weeks spec	284.	637.577	{389., 885.}	124.499	0.328285	newly 5 weeks cumul	2683.	4404.11	{3478., 5112.}	65.1956	0.214389	newly 57 weeks cumul	3203.	6407.85	{4721., 7921.}	100.058	0.291177
13	newly 3 weeks spec	574.	635.803	{424., 872.}	25.4869	0.102121	newly 3 weeks cumul	2148.	2184.62	{1605., 2796.}	18.0893	0.0808097	newly 5 weeks spec	276.	454.951	{251., 684.}	67.7714	0.198607	newly 5 weeks cumul	2826.	3175.16	{2201., 4208.}	23.0187	0.0909603	newly 54 weeks cumul	3484.	4844.85	{3145., 6818.}	43.7005	0.143593
14	newly 3 weeks spec	353.	749.145	{447., 1142.}	112.222	0.303513	newly 3 weeks cumul	1439.	2645.94	{1882., 3409.}	83.8732	0.254214	newly 5 weeks spec	163.	457.726	{235., 755.}	180.813	0.400502	newly 5 weeks cumul	1856.	3697.97	{2449., 5065.}	99.244	0.283956	newly 56 weeks cumul	2090.	4902.24	{2977., 7274.}	134.557	0.346842
15	newly 3 weeks spec	511.	686.246	{317., 1157.}	52.2686	0.171924	newly 3 weeks cumul	2278.	3031.63	{1751., 4348.}	41.8329	0.145725	newly 5 weeks spec	197.	264.138	{115., 436.}	58.8052	0.185042	newly 5 weeks cumul	2829.	3733.58	{2048., 5587.}	43.127	0.148695	newly 60 weeks cumul	3055.	4109.95	{2217., 6163.}	45.9232	0.154474
16	newly 3 weeks spec	366.	441.625	{224., 689.}	40.6421	0.143071	newly 3 weeks cumul	1694.	1765.48	{1023., 2791.}	29.9375	0.125518	newly 5 weeks spec	184.	240.984	{116., 398.}	51.3842	0.159157	newly 5 weeks cumul	2137.	2339.2	{1298., 3736.}	33.7601	0.132827	newly 56 weeks cumul	2454.	2930.14	{1594., 4596.}	39.724	0.140827
17	newly 3 weeks spec	538.	642.339	{330., 963.}	36.4312	0.143932	newly 3 weeks cumul	1689.	2313.2	{1280., 3078.}	46.8114	0.169378	newly 5 weeks spec	353.	396.373	{162., 644.}	40.7884	0.166927	newly 5 weeks cumul	2470.	3221.53	{1727., 4531.}	40.6917	0.149646	newly 56 weeks cumul	3959.	4386.12	{2057., 6660.}	32.8588	0.137465
18	newly 3 weeks spec	567.	732.967	{517., 977.}	35.0421	0.128053	newly 3 weeks cumul	1753.	2385.89	{1846., 2850.}	41.3137	0.154226	newly 5 weeks spec	394.	534.77	{322., 816.}	44.4579	0.150594	newly 5 weeks cumul	2605.	3555.31	{2592., 4440.}	39.4443	0.140565	newly 54 weeks cumul	3791.	5669.15	{3721., 7672.}	52.0953	0.170116
19	newly 3 weeks spec	442.	543.867	{310., 775.}	37.1946	0.15146	newly 3 weeks cumul	1574.	1817.13	{1179., 2344.}	28.0347	0.12639	newly 5 weeks spec	313.	398.367	{197., 666.}	47.902	0.172119	newly 5 weeks cumul	2213.	2684.43	{1649., 3715.}	33.6316	0.136439	newly 50 weeks cumul	2944.	4389.63	{2417., 6613.}	56.3349	0.178166
20	newly 3 weeks spec	441.	535.719	{372., 713.}	32.6531	0.132385	newly 3 weeks cumul	1300.	1692.78	{1330., 2136.}	37.0096	0.148006	newly 5 weeks spec	373.	448.906	{258., 670.}	40.9769	0.15638	newly 5 weeks cumul	2074.	2635.69	{1903., 3364.}	34.5784	0.134216	newly 50 weeks cumul	3655.	5043.78	{2971., 7753.}	47.3059	0.158207
21	newly 3 weeks spec	460.	779.849	{445., 1266.}	72.5938	0.213905	newly 3 weeks cumul	1920.	2939.89	{1966., 3807.}	54.8373	0.180083	newly 5 weeks spec	202.	409.767	{211., 683.}	105.676	0.269592	newly 5 weeks cumul	2437.	3922.45	{2459., 5827.}	62.8788	0.197262	newly 58 weeks cumul	2952.	4798.92	{2892., 7393.}	65.12	0.199079
22	newly 3 weeks spec	509.	630.151	{369., 973.}	39.4076	0.143833	newly 3 weeks cumul	1513.	2333.47	{1576., 3052.}	55.2283	0.17973	newly 5 weeks spec	221.	360.321	{162., 622.}	74.8399	0.205824	newly 5 weeks cumul	2121.	3173.51	{1997., 4536.}	52.5873	0.170065	newly 56 weeks cumul	2643.	3995.68	{2337., 6119.}	56.1198	0.174236
23	newly 3 weeks spec	421.	466.077	{176., 828.}	45.8103	0.190579	newly 3 weeks cumul	1389.	1787.26	{808., 2909.}	45.3818	0.164305	newly 5 weeks spec	276.	253.046	{88., 461.}	51.7057	0.25377	newly 5 weeks cumul	2005.	2387.55	{1021., 3975.}	43.5066	0.167439	newly 55 weeks cumul	2915.	2978.75	{1219., 5210.}	41.7026	0.18571
24	newly 3 weeks spec	587.	847.629	{589., 1044.}	48.3514	0.167322	newly 3 weeks cumul	2481.	2665.48	{1836., 3118.}	22.5832	0.107541	newly 5 weeks spec	276.	654.065	{401., 879.}	137.377	0.357183	newly 5 weeks cumul	3169.	4080.82	{3082., 4770.}	33.0017	0.12283	newly 56 weeks cumul	3630.	6577.76	{4570., 8941.}	81.7031	0.249642
25	newly 3 weeks spec	500.	990.274	{689., 1259.}	98.0548	0.286053	newly 3 weeks cumul	2503.	3405.21	{2723., 3997.}	39.0828	0.142088	newly 5 weeks spec	93.	603.306	{334., 1060.}	548.717	0.774701	newly 5 weeks cumul	2800.	4810.71	{3591., 5792.}	71.8111	0.229537	newly 59 weeks cumul	2905.	6255.29	{4339., 8609.}	115.328	0.32217
26	newly 3 weeks spec	372.	638.706	{257., 1125.}	84.1134	0.245642	newly 3 weeks cumul	1508.	2544.78	{1314., 3693.}	75.3316	0.231907	newly 5 weeks spec	264.	279.235	{98., 520.}	50.6313	0.221472	newly 5 weeks cumul	2071.	3248.73	{1548., 5051.}	66.8484	0.212823	newly 58 weeks cumul	2508.	3704.82	{1702., 5866.}	60.5466	0.198857
27	newly 3 weeks spec	638.	974.661	{640., 1338.}	54.371	0.17679	newly 3 weeks cumul	2129.	3359.95	{2505., 3980.}	58.6426	0.19429	newly 5 weeks spec	370.	577.613	{294., 1014.}	64.6905	0.19011	newly 5 weeks cumul	2994.	4710.82	{3299., 5761.}	57.4358	0.189219	newly 59 weeks cumul	3855.	5973.53	{3836., 8082.}	55.7345	0.179556
28	newly 3 weeks spec	519.	928.742	{685., 1175.}	80.3655	0.248468	newly 3 weeks cumul	1948.	3106.35	{2347., 3606.}	63.6948	0.217295	newly 5 weeks spec	219.	620.629	{375., 977.}	183.392	0.421368	newly 5 weeks cumul	2468.	4494.52	{3542., 5367.}	82.7195	0.256414	newly 58 weeks cumul	2735.	6263.77	{4571., 8529.}	129.023	0.348668
29	newly 3 weeks spec	430.	813.033	{509., 1157.}	90.1296	0.255376	newly 3 weeks cumul	2046.	3403.15	{2411., 4333.}	66.9808	0.213412	newly 5 weeks spec	157.	353.377	{189., 556.}	125.916	0.31201	newly 5 weeks cumul	2479.	4305.54	{2927., 5761.}	74.3617	0.228566	newly 60 weeks cumul	2601.	4890.69	{3206., 6728.}	88.6349	0.259522
30	newly 3 weeks spec	581.	999.347	{629., 1503.}	73.0098	0.218778	newly 3 weeks cumul	2678.	3665.84	{2757., 4419.}	38.2235	0.135507	newly 5 weeks spec	176.	512.467	{269., 938.}	191.174	0.41746	newly 5 weeks cumul	3250.	4916.	{3473., 6385.}	51.7276	0.171596	newly 60 weeks cumul	3417.	5914.32	{4000., 8259.}	73.1858	0.223893
31	newly 3 weeks spec	369.	424.706	{159., 764.}	49.7848	0.202841	newly 3 weeks cumul	1285.	1914.45	{807., 3250.}	63.7644	0.20755	newly 5 weeks spec	236.	177.51	{56., 352.}	50.4071	0.299488	newly 5 weeks cumul	1779.	2371.2	{962., 4109.}	53.802	0.189548	newly 57 weeks cumul	2329.	2649.78	{1025., 4566.}	45.3666	0.185558
32	newly 3 weeks spec	297.	416.344	{192., 672.}	56.1351	0.1877	newly 3 weeks cumul	969.	1467.95	{740., 2202.}	59.8454	0.190019	newly 5 weeks spec	208.	282.426	{118., 478.}	63.8083	0.221275	newly 5 weeks cumul	1426.	2093.02	{1142., 3190.}	57.9036	0.18761	newly 50 weeks cumul	2189.	3167.82	{1436., 5179.}	62.71	0.201138
33	newly 3 weeks spec	452.	505.121	{259., 748.}	35.2113	0.152872	newly 3 weeks cumul	1482.	1678.78	{961., 2305.}	30.903	0.138013	newly 5 weeks spec	292.	380.931	{198., 618.}	51.24	0.181567	newly 5 weeks cumul	2155.	2502.28	{1338., 3569.}	34.4396	0.14203	newly 50 weeks cumul	3095.	4310.03	{2397., 6887.}	53.1536	0.177102
34	newly 3 weeks spec	392.	543.867	{310., 775.}	48.4099	0.17796	newly 3 weeks cumul	1312.	1817.13	{1179., 2344.}	44.8044	0.170869	newly 5 weeks spec	229.	398.367	{197., 666.}	80.262	0.228393	newly 5 weeks cumul	1839.	2684.43	{1649., 3715.}	51.9612	0.182133	newly 50 weeks cumul	2740.	4389.63	{2417., 6613.}	64.9647	0.196771
35	newly 3 weeks spec	441.	749.952	{447., 1127.}	71.242	0.213575	newly 3 weeks cumul	1805.	2649.34	{1882., 3409.}	47.7571	0.161051	newly 5 weeks spec	239.	456.806	{239., 755.}	93.4269	0.245301	newly 5 weeks cumul	2353.	3700.55	{2449., 5054.}	58.41	0.18684	newly 57 weeks cumul	2899.	4900.68	{2977., 7108.}	70.0675	0.209789
36	newly 3 weeks spec	507.	666.766	{488., 856.}	34.4582	0.122864	newly 3 weeks cumul	1570.	2150.41	{1669., 2577.}	39.2178	0.141253	newly 5 weeks spec	332.	512.859	{309., 816.}	58.3443	0.180018	newly 5 weeks cumul	2346.	3257.91	{2444., 4060.}	39.8005	0.139402	newly 53 weeks cumul	3516.	5403.	{3424., 7797.}	55.2101	0.17517
37	newly 3 weeks spec	390.	661.714	{321., 1063.}	77.3901	0.232554	newly 3 weeks cumul	1418.	2349.43	{1200., 3174.}	74.3452	0.243136	newly 5 weeks spec	278.	429.536	{185., 814.}	72.0581	0.220857	newly 5 weeks cumul	2082.	3323.21	{2001., 4722.}	64.236	0.202728	newly 56 weeks cumul	2812.	4641.34	{2389., 8167.}	72.4427	0.218812
38	newly 3 weeks spec	545.	801.438	{538., 1084.}	50.4014	0.170554	newly 3 weeks cumul	1921.	2719.2	{1893., 3292.}	44.7984	0.158256	newly 5 weeks spec	367.	532.156	{301., 863.}	52.1373	0.166387	newly 5 weeks cumul	2763.	3918.02	{2785., 4819.}	43.9078	0.152626	newly 56 weeks cumul	3774.	5567.59	{3701., 7659.}	50.6409	0.167991
39	newly 3 weeks spec	417.	667.	{488., 856.}	60.1609	0.194932	newly 3 weeks cumul	1435.	2146.81	{1669., 2577.}	51.4376	0.176947	newly 5 weeks spec	292.	515.032	{309., 816.}	77.806	0.225336	newly 5 weeks cumul	2099.	3257.27	{2444., 4060.}	55.2544	0.183633	newly 52 weeks cumul	3353.	5417.26	{3424., 7797.}	62.3986	0.193351
40	newly 3 weeks spec	501.	599.413	{399., 843.}	30.2569	0.113637	newly 3 weeks cumul	1605.	1997.49	{1465., 2539.}	28.4458	0.105031	newly 5 weeks spec	314.	441.667	{249., 763.}	51.3649	0.16382	newly 5 weeks cumul	2336.	2956.83	{2029., 3957.}	31.9145	0.115296	newly 52 weeks cumul	3452.	4683.06	{2789., 7168.}	43.0829	0.142939
41	newly 3 weeks spec	586.	937.958	{553., 1468.}	64.8152	0.199164	newly 3 weeks cumul	2370.	3563.42	{2575., 4487.}	51.7389	0.17387	newly 5 weeks spec	259.	441.07	{203., 729.}	80.9669	0.230334	newly 5 weeks cumul	2995.	4667.69	{3183., 6385.}	57.7587	0.187291	newly 60 weeks cumul	3338.	5450.96	{3558., 7750.}	65.3651	0.203578
42	newly 3 weeks spec	574.	601.034	{386., 788.}	26.4027	0.126335	newly 3 weeks cumul	2136.	1906.12	{1318., 2358.}	17.381	0.11267	newly 5 weeks spec	240.	484.517	{275., 729.}	102.845	0.281278	newly 5 weeks cumul	2747.	2938.45	{1900., 3738.}	24.3614	0.113138	newly 51 weeks cumul	3203.	5391.09	{3216., 7594.}	69.9877	0.21102
43	newly 3 weeks spec	451.	769.383	{417., 1264.}	74.1205	0.216356	newly 3 weeks cumul	1975.	3037.38	{1934., 4021.}	55.4388	0.182279	newly 5 weeks spec	196.	368.483	{166., 678.}	93.2568	0.236581	newly 5 weeks cumul	2487.	3956.92	{2359., 5933.}	61.3825	0.194215	newly 59 weeks cumul	2787.	4637.52	{2631., 7225.}	68.7029	0.20806
44	newly 3 weeks spec	403.	648.393	{442., 882.}	64.4795	0.207544	newly 3 weeks cumul	1374.	2158.11	{1542., 2734.}	63.5109	0.217735	newly 5 weeks spec	237.	490.934	{271., 796.}	108.072	0.289595	newly 5 weeks cumul	1901.	3219.52	{2253., 4143.}	71.3223	0.225441	newly 53 weeks cumul	2836.	5245.31	{3214., 8074.}	85.4748	0.246889
45	newly 3 weeks spec	487.	749.952	{447., 1127.}	57.4452	0.181286	newly 3 weeks cumul	1897.	2649.34	{1882., 3409.}	41.5044	0.14352	newly 5 weeks spec	273.	456.806	{239., 755.}	72.8465	0.203746	newly 5 weeks cumul	2573.	3700.55	{2449., 5054.}	46.6758	0.156227	newly 56 weeks cumul	3089.	4900.68	{2977., 7108.}	60.9133	0.188068
46	newly 3 weeks spec	405.	436.2	{192., 652.}	35.4815	0.149186	newly 3 weeks cumul	1271.	1740.95	{888., 2432.}	46.011	0.160841	newly 5 weeks spec	319.	221.85	{84., 377.}	44.9687	0.260597	newly 5 weeks cumul	1984.	2274.18	{1116., 3192.}	32.6424	0.129434	newly 55 weeks cumul	2681.	2729.25	{1313., 4015.}	31.6524	0.139603
47	newly 3 weeks spec	463.	378.244	{208., 619.}	37.0068	0.200478	newly 3 weeks cumul	1416.	1635.27	{965., 2537.}	36.3718	0.143808	newly 5 weeks spec	259.	172.39	{86., 282.}	45.7764	0.278628	newly 5 weeks cumul	2039.	2065.24	{1191., 3242.}	34.0339	0.152356	newly 56 weeks cumul	2825.	2370.68	{1321., 3694.}	34.3976	0.181578
48	newly 3 weeks spec	614.	727.54	{567., 887.}	24.0499	0.0945223	newly 3 weeks cumul	1986.	2240.95	{1822., 2600.}	22.1487	0.0972737	newly 5 weeks spec	377.	599.413	{399., 843.}	60.1322	0.189923	newly 5 weeks cumul	2819.	3510.9	{2860., 4089.}	27.3484	0.104236	newly 53 weeks cumul	3973.	6196.48	{4381., 8258.}	56.0462	0.181929
49	newly 3 weeks spec	412.	709.296	{446., 1013.}	73.3967	0.222869	newly 3 weeks cumul	1467.	2450.58	{1754., 2981.}	67.9244	0.21805	newly 5 weeks spec	214.	467.197	{248., 866.}	119.225	0.300691	newly 5 weeks cumul	2027.	3504.38	{2386., 4681.}	73.1825	0.226717	newly 55 weeks cumul	2605.	4921.63	{3079., 7627.}	89.3347	0.25577
50	newly 3 weeks spec	434.	591.5	{319., 860.}	49.831	0.157023	newly 3 weeks cumul	2003.	2770.63	{1663., 3959.}	44.8827	0.149117	newly 5 weeks spec	156.	214.883	{107., 355.}	56.4423	0.171519	newly 5 weeks cumul	2450.	3346.67	{1978., 4841.}	44.8354	0.147538	newly 60 weeks cumul	2707.	3646.5	{2124., 5240.}	44.3332	0.14602
